# High heterogeneity of HIV-related sexual risk among transgender people in Ontario, Canada: a province-wide respondent-driven sampling survey

**DOI:** 10.1186/1471-2458-12-292

**Published:** 2012-04-20

**Authors:** Greta R Bauer, Robb Travers, Kyle Scanlon, Todd A Coleman

**Affiliations:** 1 Epidemiology & Biostatistics, The University of Western Ontario, London, Ontario, Canada; 2 Department of Psychology, Wilfrid Laurier University, Waterloo, Ontario, Canada; 3 The 519 Church Street Community Centre, Toronto, Ontario, Canada

**Keywords:** HIV, Transgender, Transsexual, Sexual risk, Canada, Respondent-driven sampling, HIV testing

## Abstract

**Background:**

Studies of HIV-related risk in trans (transgender, transsexual, or transitioned) people have most often involved urban convenience samples of those on the male-to-female (MTF) spectrum. Studies have detected high prevalences of HIV-related risk behaviours, self-reported HIV, and HIV seropositivity.

**Methods:**

The Trans PULSE Project conducted a multi-mode survey using respondent-driven sampling to recruit 433 trans people in Ontario, Canada. Weighted estimates were calculated for HIV-related risk behaviours, HIV testing and self-reported HIV, including subgroup estimates for gender spectrum and ethno-racial groups.

**Results:**

Trans people in Ontario report a wide range of sexual behaviours with a full range of partner types. High proportions – 25% of female-to-male (FTM) and 51% of MTF individuals – had not had a sex partner within the past year. Of MTFs, 19% had a past-year high-risk sexual experience, versus 7% of FTMs. The largest behavioural contributors to HIV risk were sexual behaviours some may assume trans people do not engage in: unprotected receptive genital sex for FTMs and insertive genital sex for MTFs. Overall, 46% had never been tested for HIV; lifetime testing was highest in Aboriginal trans people and lowest among non-Aboriginal racialized people. Approximately 15% of both FTM and MTF participants had engaged in sex work or exchange sex and about 2% currently work in the sex trade. Self-report of HIV prevalence was 10 times the estimated baseline prevalence for Ontario. However, given wide confidence intervals and the high proportion of trans people who had never been tested for HIV, estimating the actual prevalence was not possible.

**Conclusions:**

Results suggest potentially higher than baseline levels of HIV; however low testing rates were observed and self-reported prevalences likely underestimate seroprevalence. Explicit inclusion of trans people in epidemiological surveillance statistics would provide much-needed information on incidence and prevalence. Given the wide range of sexual behaviours and partner types reported, HIV prevention programs and materials should not make assumptions regarding types of behaviours trans people do or do not engage in.

## Background

Previous reports on the health of trans (transgender, transsexual or transitioned; see Table 1 for definitions) people suggest this community is disproportionately affected by HIV and other sexually transmitted infections [1-5]. HIV prevalence estimates range widely, varying from 11% to 86% among male-to-females (MTFs) in studies included in a U.S. meta-analysis; summary prevalence measures were 27.7%, based on studies where testing information was available, and 11.8% when using self-report data [3]. In a recent international meta-analysis of 25 studies from 14 countries [6], overall HIV prevalence was 27.3% in MTF sex workers versus 14.7% in MTFs not engaged in sex work, 15.1% in cisgender (non-trans) male sex workers, and 4.5% in cisgender female sex workers. Overall, MTF sex workers had a 4-fold increased risk of HIV infection compared with cisgender female sex workers. High prevalences have been observed even among youth. In a recent study of MTFs aged 15 to 24 in two U.S. cities, 67% of whom reported having engaged in sex work, 19% self-reported being HIV-positive [7]. While trans sex workers may have elevated risk, it is not clear that this stems directly from commercial sex. Studies have demonstrated that sexual risk among trans sex workers may come primarily from main partners rather than commercial partners [8,9].

**Table 1:** Key terminology

	
Core gender	one’s individual and core sense of being male or female, both or neither
Cisgender	refers to individuals whose gender identity is consistent with the gender they were assigned at birth
Female-to-male spectrum (FTM)	a trans man or a female-to-male transsexual or transgender person or a genderqueer person along the masculine spectrum
Gender spectrum	refers to the fact that gender occurs on a spectrum, rather than as discrete categories; an individuals’ sense of core gender may fall at varying points along that spectrum
Genderqueer persons	refers to people whose gender identities fall outside of the normative and binary female or male
Male-to-female spectrum (MTF)	a trans woman or a male-to-female transsexual or transgender person or a genderqueer person along the feminine spectrum
Medical transition status	the extent to which one has undergone a process of medically transitioning through use of hormones and/or surgeries to allow biological sex to more closely align with one’s core gender
Social transition status	the extent to which one has changed the gender in which they live their day-to-day life to better align with their core gender; may involve changing a name, using a new pronoun, and/or changing gender-specific aspects of one’s social presentation
Transgender	describes people who vary from conventionally prescribed gender norms
Transsexual	refers to a person who identifies with a gender that is “opposite to” that assigned to them at birth
Transitioned people	refers to those who identify simply as men or women with a medical history of transitioning sex, and no longer personally identify as transgender or transsexual
Trans people	an umbrella term for a diverse group of people including transsexual, transgender, transitioned, genderqueer, and some Two-Spirit people, whose gender identity or expression differs from societal norms
Two-Spirit	refers to North American Indigenous peoples who identify with elements of both male and female gender roles found in many traditional cultures; some but not all will identify along a trans spectrum

Compared to MTFs, there are few studies estimating HIV prevalence among female-to-male spectrum (FTM) trans people. Estimates range from 0% to 3% [3]. The only study to date to present test-based HIV seroprevalence reported that 2% of FTMs in a San Francisco sample were HIV positive, versus 35% of MTFs [1].

In the U.S., higher prevalences of HIV have been identified for African-American MTFs, with seroprevalences as high as 63% [1]. Summary measures from a meta-analysis estimate self-reported HIV positivity at 30.8% for African-American MTFs, and seroprevalence at 56.3% [3]. Considering historical and contemporary differences between the U.S. and Canada with regard to colonialism, slavery, immigration policies and patterns, and human rights policy, it is not clear to what extent ethno-racial inequities observed in the U.S. may apply in Canada. However, given the impact of experiences of racism, HIV vulnerability among racialized groups of trans people in Canada is a concern. Despite long-standing recognition of HIV vulnerabilities among Aboriginal Canadians, concerns regarding HIV among Aboriginal trans people have only begun to be addressed. Aboriginal people represented 3.8% of the Canadian population, 8% of all prevalent HIV infections, and 12.5% of all new infections in 2008 [10].

Studies have described behavioural sexual risk factors for HIV among MTFs in particular. These factors include compulsive sexual behaviour [11], sex work [11,12], multiple sex partners [1], unprotected receptive anal sex [13], and sex under the influence of drugs or alcohol [13]. Having sex with cisgender men who have sex with men [14], or having a partner of an unknown HIV status [15], were also documented risk factors for HIV.

As with prevalence estimates, for FTMs there is less information available about HIV-related sexual risk behaviours, though research suggests that some FTMs engage in high-risk sex, in particular those who have sex with cisgender male partners. Several small- to moderate-size studies report high proportions of FTMs engaging in high-risk sex, including unprotected receptive genital and anal sex [1,16-18]. Among 22 FTMs participating in an Ontario study of gay, bisexual and other men who have sex with men (MSM), about one third reported unprotected receptive anal sex in the past 6 months with a partner who was HIV-positive or of unknown status [19]; similar findings were recently reported among trans MSM in one U.S. region [20]. While most research on trans sex workers focuses on MTFs, FTMs also engage, or have engaged in sex work. One of the earliest and largest studies of FTMs, in San Francisco, found that 31% of FTM participants had a history of sex work or survival sex [1].

This emerging body of evidence on HIV risk among trans people documents extraordinarily high rates of HIV within segments of trans communities, in particular among African-American MTFs, and MTF sex workers internationally. To date, the majority of studies looking at HIV and HIV-related sexual risk behaviour have relied almost solely on convenience or venue-based samples from jurisdictions outside of Canada, and are largely based on MTFs living in inner cities or large urban centres, involved in sex work and/or accessing services where they are more likely to come into contact with researchers. While HIV prevalence among trans people is commonly assumed to be high, very little is known about HIV infection rates, testing rates, sexual risk behaviours, and even the socio-demographic structure of trans communities more broadly, including those outside of urban centres and not closely affiliated with organized trans communities. This information is essential both to more accurately identify those groups within trans communities that are at highest risk for HIV, and to avoid extrapolating from very high-risk subgroups to all trans people, some of whom will be at no risk. In addition, little is known about how socio-demographics and HIV-related factors may vary in a Canadian context, which differs significantly, for example, from that of the United States. Socio-demographics are important, as proximal sexual risk determinants (e.g. specific sexual behaviours, sex partner numbers) can be better understood in the context of the social determinants of health. This paper seeks to describe socio-demographics, self-reported HIV prevalence and HIV-related sexual risk among trans people in Ontario, Canada’s most populous province.

## Methods

### Trans PULSE Project

The Trans PULSE Project is a community-based research study of the health and HIV vulnerability of trans people in Ontario. The project involves a long-standing partnership between community agencies, academic researchers, and unaffiliated trans community members. A qualitative first phase was conducted, and the results were used to inform the development of the survey, as well as to develop and refine a theoretical model of how structural and informational barriers to health care access are created for trans people [21]. Research Ethics Board approvals were obtained from The University of Western Ontario and Wilfrid Laurier University. Results presented herein represent findings from the quantitative second phase of the project.

### Sampling

Trans participants (n = 433) were recruited using respondent-driven sampling, a method of chain-referral sampling suited to reaching hidden populations [22,23]. Seeds, or initial participants, were 16 trans people who were diversely situated, both geographically and demographically, and who served as members of the project’s Community Engagement Team. Seeds each recruited a maximum of 3 additional participants as the first wave; these similarly recruited the second wave of participants, and so on. New potential recruits received a coupon containing eligibility criteria and information regarding the study, the different modes they could choose for participation, and how to initiate the process on our survey website or via our toll-free telephone line. Upon contact, as well as through promotional materials, they were told the survey should take between 60 and 90 minutes to complete, and that the purpose of the study was to understand the health of trans people in Ontario. Once 4 to 5 waves of participants were recruited, the number of waves typically needed to obtain equilibrium (a stable sample composition through successive waves), 22 additional seeds were added. Recruitment continued from May, 2009 through April, 2010, reaching a total of 10 waves. While participants could remain anonymous, recruitment patterns were tracked using ticket numbers, and the degree (number of other eligible people known) was assessed for each participant. To be eligible, participants had to 1) indicate they were trans; 2) live, work, or receive health care in Ontario, and; 3) be age 16 or older. Trans was defined inclusively, and it was made explicit to potential participants that they were not required to have begun a social gender transition or to have undertaken interventions to medically transition sex. This broad definition of trans was made clear on recruitment coupons, the project website, the letter of information and consent, and the eligibility questions that participants had to respond to (online or via telephone) prior to participating. Participants received a $20 gift card honorarium, or could donate it to a trans-related community group; about half of honoraria were accepted and half donated. Surveys could be completed online, via a visually identical paper-and-pencil survey, or over the telephone with language interpretation. In the final two months of the study, $5 secondary incentives were added for recruiting peers, including retroactively for participants for whom we had contact information; these did not appear to impact recruitment.

### Measures

Measures included demographics, sexual behaviour history and sexual risk measures, HIV testing history, and self-reported HIV status.

#### *Demographics*

Socio-demographic measures included gender spectrum, age, ethno-racial background, region of residence, country of birth, highest educational level attained, poverty, and social and medical transition status. Gender spectrum was classified as female-to-male versus male-to-female. Not all participants identified as male or female, and these were classified by direction of gender divergence from birth sex. For example, an individual who was assigned male at birth and identified as Two-Spirit was assigned to the male-to-female spectrum group. Ethno-racial background(s) were assessed with a check-all-that-apply question, and so totals will not sum to 100%. Multiple survey items were used to categorize into the broader ethno-racial group variable for analysis. Participants who did not check “Aboriginal”, but who indicated in another item that they were First Nations, Métis, or Inuit were coded as Aboriginal. Those indicating only white background(s) were coded as white, and after data checking, the remaining individuals were coded as non-Aboriginal racialized people. In this paper, the term “racialized” is used when describing people of colour. This is consistent with usage recommended by the Ontario Human Rights Commission’s guidelines, which state that this term is “preferred over racial minority, visible minority, person of colour or non-White as it expresses race as a social construct rather than a description on perceived biological traits” [24].

Region of residence was coded based on the first letter of a participant’s postal code. Household poverty was calculated using the Statistics Canada formula for low income cut-off (LICO) applied to mid-points for household income categories. For reference, the LICO for a household of 1 (in Canadian dollars) was $21,189; for a household of 4 it was $42,378.

For social transition status, participants were asked to indicate whether they were living in their core gender full-time, part-time, or not at all. For medical transition status, they were asked their current situation regarding hormones and/or surgery, and indicated whether they had medically transitioned, were in the process, were planning to but had not begun, were not planning to, were not sure, or that the concept of “transitioning” did not apply to them. No particular medications or procedures were required to be classified as having medically transitioned, other than the participant’s indication that the process was completed for them.

#### *Sexual behaviours*

Participants self-reported information on lifetime and past-year sex partner numbers, and on past-year engagement in different types of sexual behaviours, including oral, anal and genital sex. For each, participants were asked if such activities had involved flesh genitals, silicone or latex, or fingers or hands, and how often they or their partner ejaculated without a condom.

Overall sexual risk was grouped into three categories, with high-risk sex defined according to Canadian AIDS Society guidelines [25]: 1) no sex within the past year; 2) low- to moderate-risk sex, including oral sex, or genital or anal sex without ejaculate (or ejaculation with a condom), and; 3) high-risk sex, defined as insertive or receptive genital or anal sex with fluid contact. Sex without fluid exposure also included sex where fluid exposure was to non-flesh genitals, such as penile prosthetics. Participants who engaged in “high-risk” activities, but only in the context of sex with a single HIV-seroconcordant regular partner or spouse were classified as low risk.

To assess history of sex work or exchange sex, participants were asked whether they had “ever done sex work or exchanged sex for money or other resources (e.g. shelter, drugs, food)?” Current sex work status was coded for those who indicated sex work or escort work on a multi-category item assessing type of paid work currently done.

#### *HIV testing and status*

Participants indicated whether they had ever been tested for HIV, and if so the recentness of their last test. HIV status was self-reported.

### Statistical analysis

Frequencies were estimated using Respondent-Driven Sampling Analysis Tool version 6.0 [26]. All statistical estimates presented were weighted using RDS I estimation techniques [22,27], based on the probability of recruitment, to produce estimates for the Ontario trans population. Weighting accounts for differences in network sizes, as participants who are less well connected are less likely to be recruited, as well as for differential recruitment rates across groups [27]. This adjusts for homophily, the tendency of people to know, and thus recruit, people who are more like themselves. A modified bootstrapping approach was used to construct 95% confidence intervals [23,28], with 10,000 resamples through recruitment chains. The enhanced data-smoothing algorithm was applied [23].

While statistical tests designed for random samples cannot be conducted using networked RDS data, variance recovery methods make possible the testing for statistical significance of differences between two proportions. Zou and Donner’s MOVER method was used to construct confidence intervals around the difference in proportions [29]. Where derived confidence intervals excluded 0, differences in frequencies were determined to be statistically significantly at *p* < 0.05.

## Results

**Figure 1:**
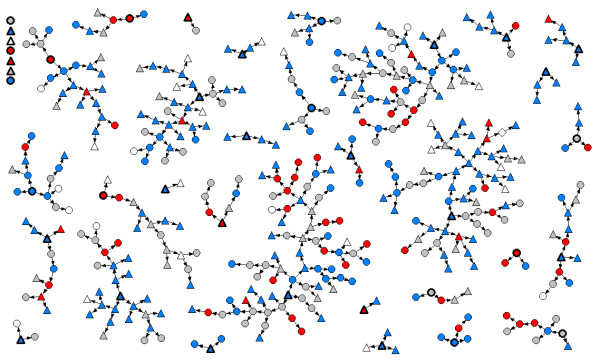
**Recruitment networks for trans study participants in Ontario, Canada (n = 433).** Triangle = female-to-male spectrum (FTM).Circle = male-to-female spectrum (MTF). Grey = no sexual risk in past year (no sex). Blue = only low-risk sex in past year. Red = high-risk sex in past year.

A total of 433 trans Ontarians participated in the study. A network diagram showing the recruitment structure of the sample is presented in Figure 1, coded for MTF versus FTM gender spectrum and overall HIV-related sexual risk. Maximum recruitment chain length was 10 waves beyond the initial seeds.

Socio-demographic results are presented in Table 2. Estimates for age distribution showed a young population of trans Ontarians, with 33% aged 16–24 years, and only 9% over age 55. An estimated 33% of trans people lived in metropolitan Toronto. The geographic distribution of trans Ontarians around the province was similar for FTMs and MTFs with the exception of Toronto and the surrounding south central Ontario region. While a similar proportion lived in the combined regions, FTMs were more likely to live in Toronto than the surrounding area. Non-Aboriginal racialized trans people were significantly more likely to live in metropolitan Toronto than were non-Aboriginal white trans Ontarians. Among non-Aboriginal racialized trans people, about two-thirds (62%) were born outside of Canada; most lived in Toronto (65%) and Central Ontario (19%), and were highly educated, reflecting general population patterns for this group. We did not find evidence of depressed income levels among non-Aboriginal racialized trans people relative to white trans people, as incomes were low across all groups. An estimated 49% of trans people earned less than $15,000 per year, and 34% lived in poverty, with household income that fell below the Statistics Canada low-income cut-off based on household size.

**Table 2:** Demographics: Weighted frequencies for trans people in Ontario, Canada

	**All trans people n = 433**		**Female-to-Male spectrum n = 227**		**Male-to-Female spectrum n = 205**		**Aboriginal n = 35**		**Non-aboriginal white n = 333**		**Non-aboriginal racialized n = 62**	
	**%**	**95% CI**	**%**	**95% CI**	**%**	**95% CI**	**%**	**95% CI**	**%**	**95% CI**	**%**	**95% CI**
**Gender spectrum**												
Female-to-male	53	(45, 62)	n/a	n/a	n/a	n/a	46	(19, 72)	49	(41, 61)	85	(71, 94)
Male-to-female	47						54	(28, 81)	51	(39, 59)	15	(6, 29)
**Age**												
16–24	33	(25, 43)	42	(30, 54)	25	(15, 38)	50	(19, 71)	34	(25, 45)	35	(19, 54)
25–34	29	(23, 37)	32	(22, 42)	27	(18, 39)	32	(16, 60)	29	(23, 39)	33	(10, 51)
35–44	16	(11, 23)	14	(7, 22)	18	(10, 28)	12	(1, 27)	14	(9, 21)	23	(8, 41)
45–54	13	(7, 18)	10	(3, 19)	15	(7, 22)	6	(0, 18)	13	(7, 19)	9	(0.2, 28)
55–64	6	(2, 10)	3	(0, 8)	10	(3, 16)	0.2	(0, 1)	7	(2, 10)	0.8	(0, 5)
65 +	3	(0.6, 5)	0	(−−, --)	6	(1, 11)	0.3	(−−, --)	3	(0.6, 6)	0.1	(−−,--)
**Ethno-racial group**												
Aboriginal	7	(4, 11)	6	(2, 11)	8	(4, 15)	n/a	n/a	n/a	n/a	n/a	n/a
Non-Aboriginal white	77	(71, 84)	70	(61, 81)	87	(80, 93)						
Non-Aboriginal racialized	16	(10, 22)	25	(14, 32)	5	(2, 9)						
Ethno-racial background(s) indicated *												
Aboriginal	6	(3, 10)	5	(2, 10)	7	(2, 12)	n/a	n/a	n/a	n/a	n/a	n/a
White Can/Amer/Euro	88	(83, 93)	84	(76, 92)	93	(87, 98)						
East/South/Southeast Asian	7	(3, 12)	10	(4, 19)	4	(1, 7)						
Black Can/Amer/African	4	(0.9, 7)	6	(1, 12)	0	(−−, --)						
Latin American	3	(0.8, 6)	4	(0.8, 9)	1	(0, 4)						
Middle Eastern	4	(1, 7)	6	(2, 12)	0.4	(0, 1)						
Other	4	(0.9, 7)	6	(0.6, 11)	2							
**Region of residence**												
Southeastern Ontario	15	(7, 25)	13	(5, 23)	16	(6, 27)	5	(0, 21)	16	(7, 28)	5	(0, 16)
South central Ontario	17	(11, 25)	9	(3, 18)	25	(16, 38)	26	(9, 54)	16	(11, 27)	19	(2, 41)
Metropolitan Toronto	33	(22, 42)	46	(30, 59)	23	(14, 32)	50	(11, 73)	30	(18, 37)	65	(42, 85)
Southwestern Ontario	27	(17, 39)	27	(15, 41)	25	(14, 40)	10	(0, 27)	30	(18, 42)	8	(0, 26)
Northern Ontario	8	(3, 16)	6	(1, 15)	10	(3, 19)	9	(1, 25)	9	(3, 18)	2	(0, 9)
**Place of birth**												
Canada	81	(75, 87)	79	(69, 89)	84	(76, 91)	91	(71, 100)	90	(84, 94)	38	(20, 58)
Outside of Canada	19	(13, 26)	21	(12, 31)	17	(9, 24)	9	(0, 29)	11	(6, 16)	62	(42, 81)
**Education**												
Less than high school	13	(8, 19)	14	(8, 25)	11	(5, 19)	15	(3, 30)	15	(9, 21)	2	(0, 7)
High school diploma	16	(11, 22)	21	(12, 28)	12	(5, 19)	18	(4, 37)	16	(10, 22)	18	(5, 32)
Some college or university	28	(22, 35)	25	(16, 34)	31	(22, 42)	34	(11, 61)	29	(22, 37)	25	(13, 43)
College or university degree	36	(28, 43)	32	(23, 42)	40	(30, 50)	28	(6, 53)	35	(27, 43)	40	(20, 59)
Graduate or professional degree	8	(4, 12)	9	(2, 15)	7	(2, 11)	6	(0, 19)	6	(2, 10)	15	(0.4, 33)
**Personal annual income**												
< $15,000	49	(41, 59)	52	(42, 65)	47	(37, 63)	61	(31, 85)	49	(41, 61)	48	(29, 73)
$15,000 - $29,999	21	(15, 29)	22	(13, 32)	21	(10, 31)	5	(0, 14)	22	(14, 31)	24	(5, 46)
$30,000 - $49,999	16	(9, 20)	20	(11, 28)	10	(4, 15)	6	(0.5, 17)	16	(9, 22)	18	(4, 29)
$50,000 - $79,999	7	(3, 11)	3	(0.7, 5)	12	(4, 20)	20	(3, 45)	7	(3, 12)	0.4	(0, 1)
$80,000 +	7	(3, 13)	4	(0.1, 10)	11	(3, 20)	8	(0, 23)	6	(2, 11)	10	(0, 27)
**Household poverty**												
Below low income cut-off (LICO)	34	(27, 42)	37	(27, 47)	31	(22, 42)	53	(21, 77)	35	(27, 45)	28	(13, 47)
Above LICO	66	(58, 73)	63	(53, 73)	69	(58, 78)	47	(23, 80)	65	(55, 73)	72	(53, 87)
**Social transition status**												
Full-time in core gender	48	(41, 57)	49	(38, 61)	46	(37, 59)	43	(23, 79)	49	(42, 59)	44	(24, 63)
Part-time in core gender	30	(22, 36)	35	(24, 47)	24	(13, 30)	38	(7, 60)	28	(19, 35)	35	(20, 53)
Not living in core gender	22	(16, 30)	16	(7, 25)	30	(21, 42)	19	(0, 40)	23	(15, 31)	21	(6, 38)
**Medical transition status**												
Completed a medical transition	25	(17, 32)	25	(15, 35)	25	(19, 39)	34	(10, 64)	24	(16, 31)	28	(11, 45)
In process	24	(19, 31)	16	(10, 22)	32	(24, 44)	41	(13, 69)	24	(18, 31)	11	(4, 20)
Planning, but not begun	27	(21, 35)	38	(28, 49)	15	(7, 22)	14	(2, 33)	30	(24, 41)	23	(7, 40)
Not planning to medically transition	4	(1, 9)	6	(0.6, 14)	3	(1, 5)	0.7	(−−, --)	5	(2, 10)	0.6	(0, 2)
Not sure	9	(5, 17)	6	(2, 15)	13	(5, 21)	4	(0, 12)	9	(5, 15)	17	(0, 36)
Concept of transitioning does not apply	10	(5, 13)	9	(4, 14)	11	(3, 15)	6	(0, 14)	8	(3, 11)	20	(9, 39)

* Ethno-racial background was a check-all-that-apply item, so totals will not sum to 100%.

MTFs were less likely to be living in their core gender, even part-time, than were FTMs. While the proportion of trans people that had completed a medical transition was similar between gender spectrums, a higher proportion of MTFs were in the process of medically transitioning, whereas a higher proportion of FTMs were planning to medically transition, but had not begun. Though the three ethno-racial groups did not differ with regard to living in their core gender full-time, part-time or not at all, they were quite different with regard to medical transition status. In particular, while the proportion having completed (by one’s own definition) a medical transition was again similar across groups, non-Aboriginal racialized trans people were significantly less likely than each of the other groups to be in the process of medically transitioning (11% vs. 41% for Aboriginal and 24% for white), and more likely to have reported that the concept of transitioning does not apply to them (20% vs. 6% and 8%).

Demographics for Ontario trans people were similar to Ontario data from the 2006 Canadian Census with regard to ethno-racial group, education, region of residence, and birth in or outside of Canada (census data not shown). However, trans people were younger than the census population, and had lower personal annual incomes. Moreover, while overall ethno-racial distributions were similar to the Ontario population, our data indicated greater ethno-racial diversity among FTMs than among MTFs.

Sexual behavioural data are presented in Table 3. Over the lifetime and in the past-year timeframes, trans people were highly heterogeneous with regard to sex partner numbers and types, as well as for the types of sex they engaged in. The majority of trans people in all groups were not at high risk for sexually acquired HIV within the past year. A high proportion did not have any past-year sex partners: an estimated 25% of FTMs and a significantly higher 51% of MTFs. This factor contributed to differences in HIV-related sexual risk profiles by gender spectrum, with MTFs being more likely to be at no past-year risk due to a lack of partner sex, but also significantly more likely to report high-risk sex. An estimated 19% of MTFs versus 7% of FTMs reported sex in the past year that was classified as high risk. The most common specific risk behaviours were receptive genital sex for FTMs and insertive genital sex for MTFs. FTMs were significantly more likely to report having sex while drunk or high in the past year than MTFs (42% vs. 22%). MTFs and FTMs were similar in their historic and current engagement in sex work. Within each group about 15% had ever engaged in sex work or exchange sex, and 2% reported current employment as a sex worker or escort.

**Table 3:** Sexual Behaviours: Weighted frequencies for trans people in Ontario, Canada

	**All trans people n = 433**		**Female-to-Male spectrum n = 227**		**Male-to-Female spectrum n = 205**		**Aboriginal n = 35**		**Non-aboriginal white n = 333**		**Non-aboriginal racialized n = 62**	
	**%**	**95% CI**	**%**	**95% CI**	**%**	**95% CI**	**%**	**95% CI**	**%**	**95% CI**	**%**	**95% CI**
**Sex partner number, lifetime**												
0	14	(10, 25)	16	(6, 24)	15	(8, 25)	14	(0, 26)	14	(10, 25)	19	(4, 40)
1	13	(6, 19)	19	(8, 30)	10	(3, 16)	4	(0, 14)	13	(6, 19)	18	(4, 32)
2–4	19	(11, 24)	18	(11, 21)	23	(13, 31)	24	(9, 61)	19	(11, 24)	16	(3, 30)
5–9	20	(15, 30)	13	(6, 20)	22	(17, 36)	6	(0, 15)	20	(15, 30)	8	(2, 19)
10–19	17	(11, 25)	23	(14, 32)	12	(4, 18)	31	(0, 60)	17	(11, 25)	22	(5, 44)
20–49	8	(4, 12)	7	(4, 12)	9	(3, 15)	15	(4, 30)	8	(4, 12)	7	(1, 17)
50 +	8	(3, 12)	6	(2, 11)	10	(3, 17)	6	(0, 15)	8	(3, 12)	11	(0.2, 23)
**Sex partners, lifetime**												
Trans men	18	(13, 26)	18	(10, 25)	9	(4, 14)	5	(0, 15)	18	(13, 26)	8	(3, 19)
Non-trans men	42	(32, 50)	45	(34, 57)	40	(29, 52)	54	(26, 79)	42	(32, 50)	46	(24, 67)
Trans women	16	(9, 22)	11	(5, 19)	19	(11, 29)	14	(4, 29)	16	(9, 22)	15	(3, 31)
Non-trans women	72	(62, 80)	66	(52, 77)	68	(55, 79)	45	(22, 70)	72	(62, 80)	54	(32, 76)
Genderqueer persons	25	(18, 35)	32	(21, 42)	12	(6, 20)	16	(4, 33)	25	(18, 35)	24	(10, 41)
**Sex partner number, past yr**												
0	39	(33, 51)	25	(15, 36)	51	(40, 64)	35	(12, 64)	39	(33, 51)	22	(7, 39)
1	35	(26, 43)	43	(31, 54)	26	(15, 36)	24	(3, 55)	35	(26, 43)	39	(17, 59)
2–4	17	(12, 25)	20	(12, 30)	12	(6, 20)	9	(0.7, 23)	17	(12, 25)	13	(4, 30)
5 +	8	(3, 11)	12	(5, 21)	11	(4, 19)	31	(3, 63)	8	(3, 11)	26	(6, 49)
**Sex partners, past yr**												
Trans men	8	(4, 13)	10	(5, 16)	4	(1, 7)	3	(0, 11)	8	(4, 13)	7	(2, 13)
Non-trans men	26	(15, 36)	21	(13, 31)	23	(13, 32)	45	(14, 67)	26	(15, 36)	23	(9, 46)
Trans women	11	(5, 16)	7	(2, 13)	14	(6, 23)	6	(0.1, 17)	11	(5, 16)	13	(2, 29)
Non-trans women	34	(26, 42)	44	(32, 53)	24	(15, 34)	14	(0.5, 34)	34	(26, 42)	39	(22, 65)
Genderqueer persons	8	(4, 14)	14	(6, 21)	3	(1, 5)	5	(0, 13)	8	(4, 14)	12	(3, 26)
Sexual behaviours, past yr *												
Received oral sex	47	(37, 56)	60	(46, 71)	37	(26, 48)	61	(32, 85)	47	(37, 56)	66	(43, 83)
Gave oral sex	51	(41, 60)	61	(48, 73)	45	(35, 57)	57	(30, 86)	51	(41, 60)	72	(55, 88)
Receptive partner in anal sex	24	(16, 30)	28	(16, 38)	29	(19, 41)	40	(13, 70)	24	(16, 30)	47	(21, 65)
Insertive partner in anal sex	18	(11, 24)	26	(16, 36)	14	(7, 22)	7	(0.1, 21)	18	(11, 24)	44	(19, 63)
Receptive partner in genital sex	40	(32, 51)	57	(46, 68)	16	(8, 27)	17	(3, 40)	40	(32, 51)	51	(28, 69)
Insertive partner in genital sex	46	(36, 55)	55	(43, 66)	32	(21, 44)	23	(3, 49)	46	(36, 55)	49	(27, 76)
Fluid-exposed sexual behaviours, past yr **												
High-risk receptive anal sex	2	(0.3, 4)	0	(0, 0.1)	4	(1, 8)	4	(0, 14)	2	(0.3, 4)	2	(0, 6)
High-risk insertive anal sex	2	(0.1, 4)	0	(−−, --)	4	(0.3, 9)	0	(−−,--)	2	(0.1, 4)	1	(0, 5)
High-risk receptive genital sex	6	(1, 11)	7	(1, 14)	3	(0, 14)	4	(0, 13)	6	(1, 11)	0.4	(0, 2)
High-risk insertive genital sex	9	(4, 15)	0.6	(0, 3)	16	(8, 26)	0	(−−,--)	9	(4, 15)	4	(0.2, 10)
**HIV-related sexual risk, past yr**												
No risk (no sex)	38	(31, 49)	25	(15, 36)	50	(39, 63)	35	(14, 61)	38	(31, 49)	19	(6, 37)
Low/moderate risk	48	(38, 57)	69	(57, 79)	31	(20, 40)	60	(34, 83)	48	(38, 57)	76	(58, 90)
High risk	14	(7, 19)	7	(1, 14)	19	(10, 30)	4	(0, 14)	14	(7, 19)	5	(1, 11)
**Had sex while drunk or high, past yr**	32	(22, 40)	42	(29, 53)	22	(13, 33)	47	(14, 73)	32	(22, 40)	39	(17, 58)
**Ever done sex work or exchange sex**	14	(8, 20)	15	(8, 23)	16	(9, 25)	35	(6, 62)	14	(8, 20)	13	(1, 32)
**Current sex worker**	3	(0.2, 6)	2	(0, 7)	2	(0.4, 4)	8	(0.1,19)	3	(0.2, 6)	0.2	(0,0.6)

* Genital and anal sex could involve penetration with flesh genitals, prostheses or toys, or fingers or hands.

** High-risk sexual behaviours are defined as involving flesh genitals (no prostheses or toys) and fluid exposure, other than with a long-term seroconcordant monogamous partner.

There were also differences in past-year sex risk by ethno-racial group, though these primarily stem from non-Aboriginal racialized trans people being more likely to have had sex (low-risk) within the past year than non-Aboriginal white trans people; there were no significant differences in high-risk sex across ethno-racial groups.

Self-reported history of HIV testing and HIV status are presented in Table 4. Self-reported prevalences of HIV were 0.6% for FTMs (95% CI: not calculable) and 3% for MTFs (95% CI: 0, 5). The estimate for self-reported HIV prevalence appeared higher for Aboriginal trans people (17%), however the confidence interval ranged from 0% to 28% indicating little precision in this smallest group (n = 35).

**Table 4:** HIV testing and HIV status: Weighted frequencies for trans people in Ontario, Canada

	**All trans people n = 433**		**Female-to-Male spectrum n = 227**		**Male-to-Female spectrum n = 205**		**Aboriginal n = 35**		**Non-aboriginal white n = 333**		**Non-aboriginal racialized n = 62**	
	**%**	**%**	**%**	**95% CI**	**%**	**95% CI**	**%**	**95% CI**	**%**	**95% CI**	**%**	**95% CI**
**HIV testing**												
< 6 mos ago	9	(5, 13)	5	(2, 9)	16	(8, 23)	8	(0, 20)	10	(5, 14)	4	(0, 9)
6 mos to < 1 yr ago	11	(7, 17)	11	(5, 19)	11	(5, 19)	34	(13, 67)	8	(3, 12)	13	(4, 36)
1 yr to < 2 yrs ago	14	(9, 19)	15	(8, 21)	14	(6, 21)	22	(0, 41)	15	(9, 20)	5	(0, 12)
2+ yrs ago	19	(14, 26)	19	(11, 26)	18	(11, 29)	22	(7, 44)	23	(18, 34)	10	(3, 20)
Never HIV tested	46	(38, 55)	51	(40, 63)	42	(31, 54)	15	(3, 36)	44	(35, 54)	67	(46, 81)
**Self-reported HIV status**												
Positive	2	(0, 2)	0.6	(−−, --)	3	(0, 5)	17	(0, 28)	2	(0, 2)	3	(−−-, --)
Negative	75	(70, 83)	77	(68, 87)	72	(64, 86)	50	(32, 89)	74	(69, 84)	71	(63, 94)
Unsure	23	(16, 29)	21	(13, 32)	25	(15, 35)	24	(1, 55)	24	(15, 30)	23	(6, 37)
Rather not say	0.6	(0, 0.9)	0.8	(0, 2)	0.2	(0, 0.4)	9	(0, 17)	0.3	(0, 0.4)	3	(−−-, --)

The self-reported prevalence of HIV testing was low, with an estimated 46% of Ontario trans people (95% CI: 38, 55) having never been tested. While lifetime testing did not differ between FTMs and MTFs, each of the three ethno-racial groups differed significantly from the other two. Aboriginal trans people were the most likely to ever have been tested for HIV, followed by non-Aboriginal whites, with non-Aboriginal racialized people least likely to have been tested. An estimated 67% (95% CI: 46, 81) of non-Aboriginal racialized people have never been tested for HIV.

## Discussion

This study contributes significant and critical information to the literature addressing HIV-related risk in trans communities. First, the study uses respondent-driven sampling, which through design and analysis strategies minimizes biases associated with convenience sampling that are present in the published literature. The estimates of HIV-related risk behaviours are considerably lower than in studies where convenience samples were used. Trans people were also more heterogeneous with regard to sex partner numbers and types, as well as for the types of sex they engaged in, with the majority not at high risk for sexually acquired HIV within the past year. A high proportion (one quarter of FTMs and half of MTFs) did not have any past-year sex partners, contributing to low prevalences of high-risk sex. FTMs reported unprotected receptive genital sex and MTFs insertive genital sex as the most common high-risk behaviours. Unlike other studies, high-risk sex did not differ across ethno-racial groups, though HIV testing history did. Low rates of HIV testing among trans people in Ontario were reported, compared to other jurisdictions, with the lowest lifetime testing among non-Aboriginal racialized people and the highest among Aboriginal people. In addition, while the focus of previous studies suggests that sex work is largely the purview of MTFs, in this study, MTFs and FTMs did not differ in both their historic and current engagement in sex work. Finally, prevalences of HIV infection were lower compared with other studies that used convenience sampling. Given low prevalences of testing and low statistical precision, however, estimates for self-reported HIV prevalence of 0.6% for FTMs and 3% for MTFs should be interpreted with caution.

That our estimates for trans people were similar to the broad Ontario population with regard to education, region of residence, and birth within versus outside of Canada, support the success of our sampling method in reaching trans people broadly. However, trans people constitute a hidden population, and it is not known to what extent trans demographics actually mirror population demographics. To assume similarity would be to assume trans people are born at, transition at, immigrate at, and survive at rates proportionate to the population, and there are reasons to expect that this may not be true. Violence against trans people and suicide, in particular, have been recently raised as serious health and equity concerns [30-32]. These may seriously impact the survival of trans people, though little published research exists. Immigration, transition and survival may also explain, to some extent, the reduced ethno-racial diversity among MTFs in particular. However, it is also possible that these differences were created in the process of network-based data collection, through network structural factors, or differences in recruitment or participation across groups. Trans population estimates describe a population that is younger and has lower personal income than Ontarians broadly. A younger age distribution has been observed consistently across trans studies. This may in part explain low incomes, as income generally increases with age, however high levels of employment discrimination have been documented [33], and it is unlikely that low incomes are simply an age-related effect.

Existing trans-specific or trans-friendly services, while limited, are concentrated in Toronto. That two-thirds of trans people did not live in metropolitan Toronto illustrates the need for development of trans-friendly services in smaller Ontario cities and towns. A recent population-based study, using a broad definition of transgender, estimated that 0.5% of the adult Massachusetts population was trans [34]. While it is not clear how this estimate would apply to the Ontario population, as a population-based estimate it represents the best information to date. Applying this estimate to the 2008 population of 10,710,200 Ontario residents over age 15 [35] (to most closely match the 16-year age limit of our study), we would estimate that there are approximately 53,500 trans residents of Ontario, 36,000 of whom do not live in Toronto.

Self-reported HIV prevalence was estimated at 0.6% for FTMs and 3% for MTFs, higher than expected based on overall population estimates for Ontario. In 2008, there were an estimated 26,627 prevalent HIV infections in Ontario [36], for a 2008 population of 10,710,200 residents age 15 and over [35], representing an overall HIV prevalence of 0.25% or 1 in 400. Of these, it has been estimated that two-thirds, or about 0.17%, were aware of their HIV status [36]. Estimates from the current analysis are that 2% of trans people (1.7% without rounding) self-report HIV positivity, 10 times the expected baseline value. However, given the width of the confidence intervals and the high proportion of trans people that had never been tested, it is not possible to accurately estimate HIV prevalence from these survey data. As in any study, there are limitations to this analysis. While estimates from RDS have been shown to be statistically unbiased [27], confidence intervals are wide. For this reason, point estimates should not be over-interpreted, but rather interpreted with regard to the range of plausible values.

Self-reported HIV prevalence was lower than in other studies with more urban, street-active samples (e.g. 11.8% from a U.S. meta-analysis for MTFs) [3]. While existing studies point to extremely high vulnerability to HIV within segments of trans communities in some cities, our evidence did not support the existence of such high levels on a broader population basis in Ontario. Our estimates were similar to, though slightly lower than, the self-report estimates obtained in the U.S. National Transgender Discrimination Survey [31]; prevalences of HIV and other sexually transmitted infections in Canada are lower than those in the U.S, in general. Whether between-study differences reflect effects of sampling high-risk versus broad population groups of trans participants, differences related to testing, or differences between actual HIV risk and prevalence in the U.S. and other countries versus Canada is unclear. While it is not a perfect remedy, Canada has human rights protections in place for trans people (under the grounds of sex) that do not exist in many other jurisdictions. Further, within Canada, most health care services are freely available to all Canadians, with administration and delivery responsibilities falling on each province or territory. Additionally, costs of specific surgeries associated with transitioning are covered in some provinces and territories, including Ontario. Costs for prescription drugs, including hormones, are largely not covered by the public health care system, but are lower than in the U.S. It is possible that existing protections may serve to mitigate some of the serious effects of discrimination, and the health inequities they produce.

It is important to note that self-reported estimates are likely underestimates of actual HIV prevalence. About a quarter of trans people reported that they were unsure of their HIV status, and about half have never been tested. History of testing varied significantly by ethno-racial group, but not by gender spectrum. Only 15% of Aboriginal trans people had never been tested, versus 44% of non-Aboriginal white people and 67% of non-Aboriginal racialized people. While we were not able to determine why these differences may exist, it is possible that the higher testing rates in Aboriginal trans people result from inclusive campaigns targeting Two-Spirit people. Outside of Aboriginal communities, campaigns targeting MSM, for example, may not even seem relevant to trans MSM. It is also possible that the greater awareness of HIV-related issues in Canadian Aboriginal communities may also contribute to increased perception of risk by Aboriginal trans people or their health care providers.

It is surprising that HIV testing was so low, given that it is free across the province of Ontario, and anonymous testing is available in most jurisdictions. As a comparison, despite similarities in estimates of partner types, MTF vs. FTM frequencies of sex, and transition status, a survey of trans people in Virginia found that only 18% had never been tested for HIV [37], versus 46% in our study. It has been argued that Canada’s punitive HIV non-disclosure laws, in place since 1998, may deter people from testing [38]. In the context of HIV testing services, barriers to inclusion can also occur due to erasure of trans people at the informational and institutional levels [21]. Erasure is the process through which trans people, and by extension trans communities, are systematically rendered invisible through passive or active exclusion, including the assumption that information on trans people, or policies to accommodate them are not relevant [21]. As an example, for many years the largest anonymous HIV testing site in Ontario’s largest city had “men’s days” and “women’s days” and when to attend – and indeed the safety of attending – was unclear to potential users who were trans. It is not readily apparent which factors affect HIV testing, and to what extent non-testing is due to low risk of HIV versus barriers that prevent testing in moderate- to high-risk individuals.

The profile of HIV risk with regard to sexual behaviours is highly heterogeneous. In the past year, MTF individuals were both more likely to have high-risk sex and to not have partner sex at all. Population statistics indicate that about 16% of a young- to middle-age adult population will not have had sex in a given year [39]. That half of MTFs and a quarter of FTMs have not had past-year sex is likely indicative of the difficulties trans people face in finding romantic or sexual partners. Gender spectrum differences may be due to a greater difficulty for MTFs in finding good romantic or sexual partners who will see them as their core gender sexually; effects of hormonal treatments on sex drive may possibly play a contributing role. Based on the comparisons of ethno-racial groups, only non-Aboriginal racialized trans people were similar to expected population levels with regard to having past-year partner sex; an estimated 81% had sex and 19% did not. While non-Aboriginal racialized trans people were more likely to be sexually active than other trans ethno-racial groups, this did not correspond with an increase in high-risk sex or in sex while drunk or high, where proportions were similar to other groups.

While sexual behaviours—which could involve penetration with fingers, penile prostheses, or toys as well as flesh genitals—were highly varied, most did not correspond to high-risk (i.e. flesh contact and fluid exposed) activities. Indeed, the greatest contributors to HIV-related sexual risk were the two sexual activities some might assume trans people are unlikely to engage in: receptive genital sex for FTMs, and insertive genital sex for MTFs. For MTFs at least, this differs from some previous studies. For example, in one San Francisco study, of over 300 MTFs, only 2 had insertive genital sex in the prior 6 months [1].

While most research on trans sex workers focuses on MTFs, FTMs in our study were similar with regard to sex work histories, as well as current sex work. Other studies have documented high frequencies for sex work among trans men in U.S. cities [1]. It is unknown whether FTMs engaged in sex work while presenting as male or female, and whether the frequency and duration of sex work involvement is similar to MTFs.

Overall, the sexual risk profile observed for Ontario trans people is quite different from the bulk of existing studies. The breadth of the population, both geographically and demographically, may provide a broader picture of trans sexuality and health outside of urban centres. Moreover, it may be that effects of transphobic discrimination in Canada are mitigated by the existence of legal human rights protections and processes for redress, and by the social safety net to the extent that it exists. While some segments of trans communities in Ontario are at higher risk than others, we do not see evidence of uniformly high risk. Indeed, the majority of trans people were not at high risk for sexually acquired HIV in the past year.

## Conclusions

In most jurisdictions in Canada and elsewhere, epidemiologic surveillance statistics for HIV do not include trans people, despite arguments from researchers that a demographic category should be created [8]. Because data do not identify trans people, it is difficult to know to what extent they are impacted by HIV. Moreover, population health surveys that assess HIV-related issues such as sexual behaviour or substance use very rarely include measures to identify trans participants, though some surveys have recently added detailed measures [40]. Additional population-level data, in the form of surveillance statistics, probability-based population health survey data, or trans-specific studies that take a large-scale population approach are needed to provide an accurate picture of HIV and trans health more broadly.

Self-reported HIV prevalence among trans people in this broad population sample appears higher than expected based on provincial levels, similar to another broad sample in the U.S.[31], and lower than the very high prevalences observed in U.S. and international urban samples with heavy representations of street-active and sex working MTFs. Additional research is needed to understand with greater accuracy the distribution of HIV-related risk within diverse trans communities, and to develop programs and policies to protect those at highest risk. Specific information on trans people outside of urban centres, within ethno-racial communities, including Aboriginal communities, and on trans youth are needed. Moreover, qualitative or mixed-methods studies may be useful in illuminating the experiences of trans people with regard to HIV-related decision-making, testing, treatment, and prevention.

Low rates of HIV testing make our self-reported prevalence difficult to interpret with regard to true HIV prevalence. Additional research also needs to be conducted to examine reasons for the observed low testing rates among trans Ontarians, and to explore why these may differ by ethno-racial group. Trans-sensitive HIV testing and prevention programs are needed throughout the province. Trans sexuality is not easily captured in conventional ways of thinking about HIV-related risk, and our results caution against making any assumptions about the types of sex trans people have, the body parts they use, or who their sex partners are. This has implications for design of prevention and education programs.

## Abbreviations

FTM: Female-to-male gender spectrum; HIV: Human immunodeficiency virus; LICO: Low-income cut-off; MTF: Male-to-female gender spectrum.

## Competing interests

The authors declare that they have no competing interests.

## Authors’ contributions

All authors conceptualized the paper. GB conducted the analyses and drafted most of the text. All authors contributed to drafting the background, collectively interpreted the results, contributed to the writing and editing of the paper, and approved the final version. All authors read and approved the final manuscript.

## Pre-publication history

The pre-publication history for this paper can be accessed here:

http://www.biomedcentral.com/1471-2458/12/292/prepub
